# Vagrants and Disease

**Published:** 1904-11-19

**Authors:** 


### Vagrants and Disease.

The question of the effect of vagrancy in the diffusion of disease, to which we have recently had occasion to refer, was on the 10th instant made the subject of discussion at an important conference convened for the purpose, and held in the hall of the London County Council under the presidency of Mr. Jephson, the chairman of its public health committee. The conference was composed of representatives of the county councils of England and Wales, of the Corporation of the city of London, of the councils of county and metropolitan boroughs, and of the Metropolitan Asylums Board, and was attended by over two hundred gentlemen in one or other of these capacities. The chief part in the proceedings was taken by Alderman Newton, of Newcastle-upon-Tyne, a place which has had long and frequent experience of the evils which it was the object of the meeting to alleviate or prevent. Situate on the eastern highway between Scotland and London, and a shipping centre, it has on many occasions suffered severely; and Alderman Newton, after declaring that in 450 cases of small-pox the introduction of the disease had been traced to tramps, went on to say that the Newcastle epidemic of 1902-1903 had put the borough to an expense of over £10,000. He moved as resolutions:—"That the conference recognises the increasing amount of habitual vagrancy as the cause of widespread and disastrous consequences to the public health, and is of opinion that much more effective measures than are at present adopted should be taken for preventing the spread of infectious disease by vagrants and for effectually dealing with this great and growing danger," and "that the evil can only be met by conferring further powers upon the local authorities, viz., the sanitary authority, the boards of guardians, and the magistracy." The meeting appears to have been singularly free from faddists, for practically the only opposition to these and to a succession of equally practical and sensible resolutions proceeded from a gentleman from Fulham, who, on a resolution in favour of the compulsory vaccination or re-vaccination of vagrants who had been exposed to the infection of small-pox, desired to air his "disbelief" in the efficacy of vaccination as a preventive and was ruled to be out of order by the chairman. A comic element was, nevertheless, introduced into the discussion of this motion by Dr. Cameron, of Leeds, who carried an amendment to the effect that the local authority should have power to compensate persons so vaccinated for "loss of time." The value of the "time" of a vagrant would by most persons be regarded as an inappreciable quantity, but Dr. Cameron apparently regards the privilege of receiving and imparting small-pox as one which should not lightly or without compensation be surrendered. We cannot but regret the passage of this amendment. That it was carried is not altogether a matter for surprise in view of present-day social tendencies; when the fear of encroaching upon the liberty of individuals hampers every sanitary and hygienic reform. The supposition that each citizen should, as a matter of course, willingly perform any simple duties which are essential for the safety of the community in which he lives is considered altogether preposterous in this cultured and enlightened age. So we have some two hundred intelligent officials giving their endorsement to the awkward principle that a man may do wrong unless a suitable reward is offered for his virtuous behaviour. It is a pity that the mutual bond which was implied by the expression "Civis Romanus sum" has no parallel in our own country to day.

